# The Epidemiological Boehringer Ingelheim Employee Study—Part I: Impact of Overweight and Obesity on Cardiometabolic Risk

**DOI:** 10.1155/2013/159123

**Published:** 2013-08-12

**Authors:** Kerstin Kempf, Stephan Martin, Carmen Döhring, Klaus Dugi, Carolin Wolfram von Wolmar, Burkhard Haastert, Michael Schneider

**Affiliations:** ^1^West-German Centre of Diabetes and Health, Düsseldorf Catholic Hospital Group, 40591 Düsseldorf, Germany; ^2^Department of Medical Statistics, RWTH-Aachen University, 52062 Aachen, Germany; ^3^Boehringer Ingelheim Pharma GmbH, 55218 Ingelheim, Germany; ^4^Boehringer Ingelheim Pharma GmbH & Co. KG, 55218 Ingelheim, Germany; ^5^mediStatistica, 58809 Neuenrade, Germany; ^6^Medical Corporate Department, Boehringer Ingelheim Pharma GmbH & Co. KG, 55218 Ingelheim, Germany; ^7^Mannheim Institute for Public Health, Medical Faculty Mannheim, Ruprecht-Karls University Heidelberg, 68131 Mannheim, Germany

## Abstract

*Objective.* Obesity-dependent diseases cause economic burden to companies. Large-scale data for working populations are lacking. Prevalence of overweight and obesity in the Boehringer Ingelheim (BI) Employee cohort and the relationship between body mass index (BMI) and cardiometabolic risk factors and diseases were estimated. *Design and Methods.* Employees (≥38 years, employed in Ingelheim ≥2 years; *n* = 3151) of BI Pharma GmbH & Co. KG were invited by the medical corporate department to participate in intensive health checkups. Cross-sectional analysis of baseline data collected through 2006–2011 was performed. *Results.* 90% of eligible subjects participated (*n* = 2849). Prevalences of overweight and obesity were 40% and 18% and significantly higher in men and participants ≥50 years. Cardiometabolic risk factor levels and prevalences of cardiometabolic diseases significantly increased with BMI and were higher in overweight and obese participants. Cut-points for increased risk estimated from ROC curves were **≈**25 kg/m^2^ for hypertension, hypercholesterolemia, arteriosclerosis, and hypertriglyceridemia and 26.7–28.0 kg/m^2^ for the metabolic syndrome, insulin resistance, hyperinsulinemia, increased intima media thickness, and type 2 diabetes. *Conclusion.* This is the first large-scale occupational health care cohort from a single company. Cardiometabolic risk factors and diseases accumulate with increasing BMI. Occupational weight reduction programs seem to be reasonable strategies.

## 1. Introduction

Over the last two decades, the prevalence of obesity has risen worldwide. Data from epidemiological studies indicate a high percentage of obese and overweight subjects in the German population compared to other countries in Western and Middle Europe, in particular, among men [1]. As obesity is an important risk factor for a number of cardiometabolic diseases, its increasing prevalence provokes substantial health and economic burden not only to society but also to companies by generating costs for nonproductive time and early retirement [2]. However, valid and representative data about prevalence of overweight, obesity, and the connected cardiovascular risk in the German operational medicine are lacking so far. The Boehringer Ingelheim (BI) Employee occupational health care cohort should bridge this gap. The BI medical corporate department offered the prevention and health care program “FIT IM LEBEN-FIT IM JOB” with intensive health checkups for free and advices for lifestyle changes. Aim of this analysis was to estimate (1) the prevalence of overweight and obesity in the baseline cross-sectional data from the BI Employee cohort, (2) correlations between BMI and other cardiometabolic risk factors (i.e., age, blood pressure, triglycerides, HbA1, intima media thickness, and total, LDL, and HDL cholesterol), (3) levels of those cardiometabolic risk factors in case of overweight and obesity, (4) prevalence of cardiometabolic diseases (i.e., hypercholesterolemia, hypertension, metabolic syndrome, hypertriglyceridemia, insulin resistance, arteriosclerosis, hyperinsulinemia, cardiovascular diseases, type 2 diabetes mellitus, and increased intima media thickness) stratified by sex, age, and BMI, (5) risk for cardiometabolic diseases with increasing BMI, and (6) BMI cut-points for increased cardiometabolic risk.

## 2. Materials and Methods

### 2.1. Study Subjects

The BI Employee study is a working population cohort. Employees (inclusion criteria: ≥38 years; employed for at least 2 years; no exclusion criteria) of BI Pharma GmbH & Co. KG were invited by the medical corporate department to participate in the prevention and health care programme “FIT IM LEBEN-FIT IM JOB”. This program is still ongoing. BI has 11.237 employees in Germany with 5.927 eligible for participation. For the present analysis only data from employees from Ingelheim were included. From 3151 eligible employees in Ingelheim (35% with desk and 65% with heavy work) 2849 (90%) agreed to participate. For the present analysis only data from those participants joined the program between 2006 and 2011 were analyzed. First participant was enrolled on 09.01.2006, the last subject reported here on 06.09.2011. The epidemiological analysis of the study was approved by the ethics committee of the Ärztekammer Nordrhein (ID 2011340); all clinical investigations had been conducted according to the principles expressed in the Declaration of Helsinki, and participants gave written informed consent.

### 2.2. The “FIT IM LEBEN-FIT IM JOB” Health Care Program

The program offers every 3–5 years intensive health checkups for free and advices for lifestyle changes at the medical corporate department or at a cooperating practice for preventive medicine. During the visit, participants filled in self-assessment questionnaires concerning family anamnesis, persistent diseases, actual diseases, medication, status of vaccination, smoking, stress, colon carcinoma prevention, satisfaction with the “FIT IM LEBEN-FIT IM JOB” program, dietary intake, and physical activity. Within the nutrition questionnaire participants had to mark how often they consumed defined nutrition components. In parallel, it was asked for the frequency and intensity of physical activity in several categories. The following parameters had been collected with standard methods: clinical anamnesis and anthropometry (e.g., weight, body mass index, waist circumference, HbA1c, lipids, and blood pressure). Blood was collected in fasting state with the vacutainer blood collection Set (Becton Dickinson, Heidelberg, Germany) and blood laboratory parameters (i.e., glucose, insulin, triglycerides, HbA1c, and total, LDL, and HDL cholesterol) biomarkers for lipid and glucose metabolism as well as for liver and kidney function had been analyzed at Bioscientia (Ingelheim am Rhein, Germany). In addition, participants were examined by sonography (of abdomen, thyroid), color-duplex ultrasound, ultrasound determination of intima media thickness (http://www.mesa-nhlbi.org/moreinfo.aspx) of the aorta carotis communis and aorta abdominalis as well as for determination of plaques or stenoses, resting ECG, and lung function and fitness (spiroergometry and lactate). Results are given to the participants in written form and with an copy for their treating physician. According to the results of the baseline checkup, the company offers several programs concerning stress management, induction of physical activity, information about healthy nutrition, and back exercises. The programs were designed on several groups, for example, focusing on sleep, nutrition and physical activity for shift worker; focusing on nutrition and physical activity for subjects with overweight or obesity or at risk for becoming overweight or obese; focusing on physical activity and time management for subjects with hypertension or field sales employees; smoking cessation for current smokers. During the personalized consultation meetings programs were offered and adapted to the respective needs. The primary outcome of this analysis was the BMI and its relationship to cardiometabolic risk factors (i.e., age, blood pressure, triglycerides, HbA1, intima media thickness, and total, LDL, and HDL cholesterol) and diseases (i.e., hypercholesterolemia, hypertension, metabolic syndrome, hypertriglyceridemia, insulin resistance, arteriosclerosis, hyperinsulinemia, cardiovascular diseases, type 2 diabetes mellitus, and increased intima media thickness). For definition of cardiometabolic disease, see Table 3 legend.

### 2.3. Statistical Analysis

Cross-sectional analysis of baseline data was performed in order to describe the prevalence of cardiometabolic risk factors and diseases in relationship to BMI. Variables were described depending on their distribution by mean ± standard deviation, median, and frequency tables. Stratification by sex, age classes, and BMI classes was performed. Differences between subgroups were statistically tested by *t*-test, Chi-square test and ANOVA test. Associations between BMI and continuous variables were estimated by Pearson correlation. Prevalences of cardiometabolic risk factors and diseases were estimated including 95% confidence intervals (by Pearson-Clopper), overall and stratified. Different logistic regression models were fitted using each cardiometabolic risk factor or disease as dependent variable and BMI as independent variable to estimate odds ratios (ORs) with 95% confidence intervals (CIs) corresponding to 1 kg/m² change of continuous BMI, respectively, ORs of BMI classes compared to reference. Furthermore, age-sex-adjusted ORs were estimated by including age class (≥50 versus <50 years) and sex as independent variables in the models. ROC analyses were performed to visualize results from logistic regression and for derivation of reasonable cut-points with respect to risk estimation of BMI. For each ROC curve, a cut-point was derived as the point of maximal distance from the diagonal corresponding to the maximum of Youden's index [3] which is defined as *Youden's index (c) = Sensitivity (c) + Specificity (c) − 1* with *c = cut-point corresponding to the ROC curve*. If this point is chosen as a cut-point for a diagnostic test, the equally weighted sum of sensitivity and specificity is maximal. This value corresponds to a BMI value. Furthermore, the area under the curve (AUC) was estimated for each ROC analysis together with a 95% confidence interval. Statistical tests were two-sided. The level of significance was 0.05, if not stated otherwise. Statistical analyses were performed using SAS version 9.3.

## 3. Results

### 3.1. High Prevalence of Overweight and Obesity in an Occupational Health Care Cohort

**Figure 1:**
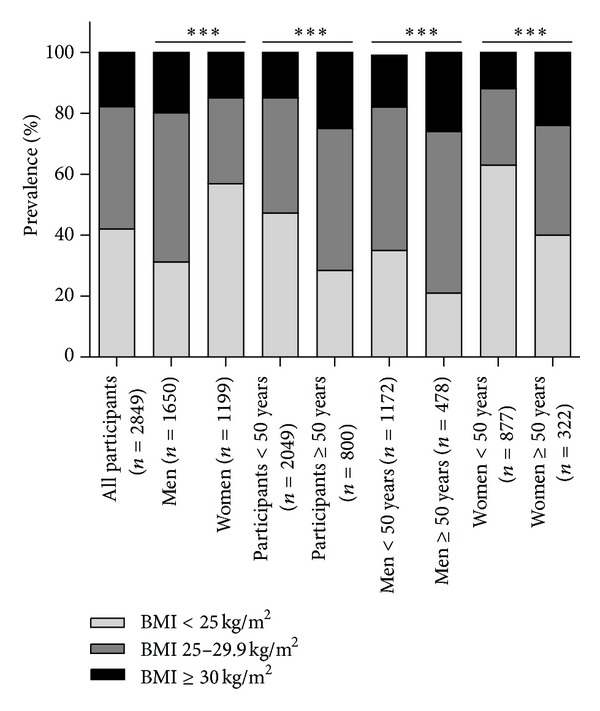
Prevalence of overweight and obesity. Normal weight was defined as BMI < 25 kg/m^2^, overweight as BMI 25–29.9 kg/m^2^ and obesity as BMI ≥ 30 kg/m^2^. Differences were determined by Chi-square test (***P < 0.001).

90% of eligible participants agreed to participate in the study. The cohort included 58% men and 42% women with a mean age of 46.4 ± 5.9 years. Generally, values for weight, BMI, waist circumference, systolic and diastolic blood pressure, triglycerides, total and LDL cholesterol, HbA1c, and intima media thickness were significantly higher in men compared to women (Table 1). The prevalence of overweight (BMI 25–29.9 kg/m^2^) was 40% and that of obesity (BMI ≥ 30 kg/m^2^) 18% (Figure 1). In men overweight (49% versus 28%) and obesity (20% versus 15%) were significantly higher compared to women (*P* < 0.001) with less than 1/3 of men (31%) but more than 1/2 of women (57%) with normal weight. Overweight (38% versus 47%) and obesity (15% versus 25%) were more pronounced in participants ≥ 50 years (*P* < 0.001) compared to those <50 years. Thus, prevalence of overweight and obesity was highest in men ≥ 50 years (53% and 26%) and lowest in women < 50 years (25% and 12%).

### 3.2. Association with BMI: Cardiometabolic Risk Factors Were Significantly Higher in Case of Overweight and Obesity

**Figure 2:**
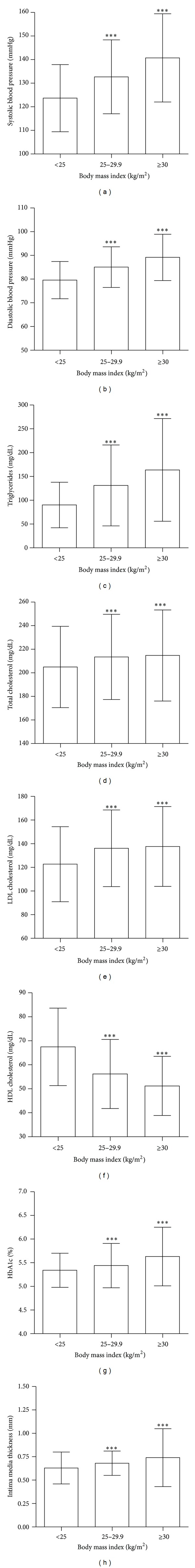
Cardiometabolic risk factors stratified by BMI. Shown are mean ± standard deviations for (a) systolic blood pressure, (b) diastolic blood pressure, (c) triglycerides, (d) total cholesterol, (e) LDL cholesterol, (f) HDL cholesterol, (g) HbA1c, and (h) intimamedia thickness stratified by BMI categories. Differences were determined by ANOVA test, log values for triglycerides (***P < 0.001).

Age, blood pressure, blood lipids, HbA1c, and intima media thickness were significantly associated with BMI (all *P* < 0.0001; Table 2). While participants with normal weight had a systolic blood pressure of 124 ± 14 mmHg and diastolic blood pressure of 80 ± 8 mmHg, mean values were significantly higher (133 ± 16 mmHg and 85 ± 9 mmHg) when participants were overweight or obese (141 ± 19 mmHg and 89 ±10 mmHg; *P* < 0.001 each; Figure 2). Also levels of triglycerides (normal weight: 90 ± 48 versus overweight: 131 ± 85 versus obesity: 164 ± 108 mg/dL; *P* < 0.001), total cholesterol (205 ± 34 versus 213 ± 36 versus 215 ± 39 mg/dL; *P* < 0.001), and LDL cholesterol (123 ± 32 versus 136 ± 32 versus 138 ± 34 mg/dL; *P* < 0.001) were significantly increased, while HDL cholesterol levels were significantly lower in overweight and obese participants (67 ± 16 versus 56 ± 14 versus 51 ± 12 mg/dL; *P* < 0.001). Also HbA1c levels (5.3 ± 0.4 versus 5.4 ± 0.5 versus 5.6 ± 0.6 mg/dL; *P* < 0.001) and intima media thickness (0.63 ± 0.17 versus 0.68 ± 0.13 versus 0.74 ± 0.31 mg/dL; *P* < 0.001) were significantly higher than those in normal weight subjects.

### 3.3. Higher Prevalence of Cardiometabolic Diseases in Males Aged ≥50 Years and Overweight or Obesity

**Figure 3:**
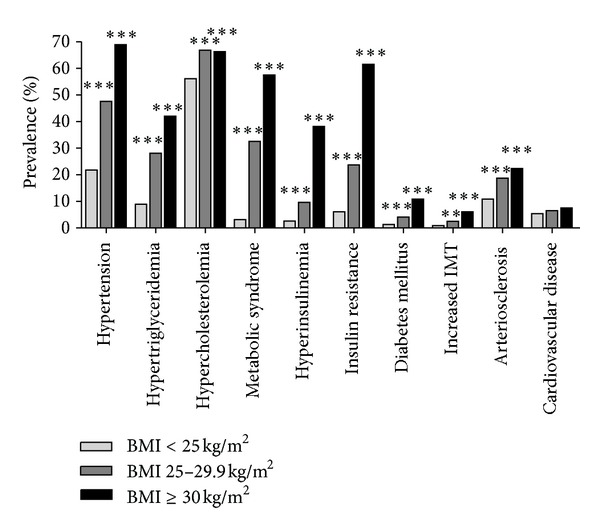
Prevalence of cardiometabolic diseases stratified by BMI. Differences statistically tested by overall likelihood ratio tests from logistic regression analysis (**P < 0.01; ***P < 0.001).

The highest prevalence of cardiometabolic diseases was observed for hypercholesterolemia (62% [95% CI 60–64%]), followed by hypertension (41% [39–43%]), metabolic syndrome (24% [22–26%]), hypertriglyceridemia (23% [21–24%]), and insulin resistance (23% [21–25%]), respectively (Table 3). Signs of manifested arteriosclerosis, that is, plaques or stenosis, were present in 16% [15–18%] of participants and hyperinsulinemia in 12% [11–13%]. Lowest prevalences were seen for cardiovascular diseases (6% [5–7%]), type 2 diabetes mellitus (4% [3–5%]), and increased intima media thickness (2% [2-3%]). Prevalences of hypertension, hypertriglyceridemia, hypercholesterolemia, metabolic syndrome, hyperinsulinemia, insulin resistance, increased intima media thickness, and arteriosclerosis were significantly higher in men compared to women (all *P* < 0.001). All cardiometabolic diseases were higher in participants ≥50 years (*P* = 0.007 for cardiovascular diseases; all other *P* < 0.001). Except for cardiovascular diseases, those prevalences were also significantly higher in overweight and obese participants (Figure 3). For instance, in the group of participants with a BMI between 25 and 29.9 kg/m^2^ (≥30 kg/m^2^), respectively, prevalences of hypertension increased from 22% (normal weight) to 48% (69%), hypertriglyceridemia from 9% to 28% (42%), hypercholesterolemia from 56% to 67% (66%), metabolic syndrome from 3% to 33% (58%), hyperinsulinemia from 3% to 10% (38%), type 2 diabetes mellitus from 1% to 4% (11%), increased intima media thickness from 1% to 2.5% (6%), and arteriosclerosis from 11% to 19% (22%).

### 3.4. Odds Ratios for Cardiometabolic Diseases Increase with BMI Increase, and Different Cut-Points for Increased Risk Were Estimated from ROC Curves

**Figure 4:**
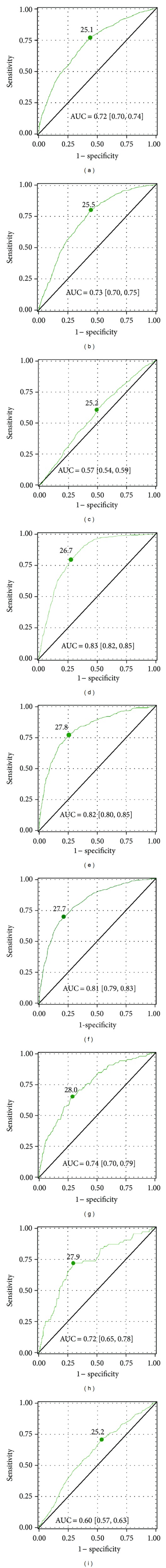
BMI cut-points for increased cardiometabolic risk. ROC analyses had been performed for (a) hypertension, (b) hypertriglyceridemia, (c) hypercholesterolemia, (d) metabolic syndrome, (e) hyperinsulinemia, (f) insulin resistance, (g) type 2 diabetes mellitus, (h) increased intima-media thickness (IMT), and (i) arteriosclerosis. Cut-points in maximal distance from the diagonal and the area under the curve [95% CI] were calculated as described in the statistical methods.

Odds ratios for hypertension, hypertriglyceridemia, hypercholesterolemia, metabolic syndrome, hyperinsulinemia, insulin resistance, type 2 diabetes mellitus, increased intima media thickness, and arteriosclerosis significantly rose with continuous or classified BMI (Table 4). Compared to normal weight subjects, participants with overweight and obesity had significantly higher odds ratios for almost all cardiometabolic diseases. In detail, highest BMI dependency was seen for insulin resistance, the metabolic syndrome and hyperinsulinemia followed by hypertension, type 2 diabetes mellitus, hypertriglyceridemia, and increased intima media thickness. Only low impact for BMI was seen for the risk of arteriosclerosis and hypercholesterolemia, while no significant dependency was found for cardiovascular diseases. Indeed, for none of the parameters, that is, angina pectoris, coronary stenoses, myocardial infarction (after age, sex adjustment), cardiac arrhythmia, heart failure, stroke, or TIA that had been used to define cardiovascular disease, a significant BMI dependency was observed. Results remained stable after adjustment to sex and age. ROC analyses were performed to visualize results from logistic regression and for derivation of reasonable cut-points with respect to risk estimation of BMI (Figure 4). The lowest cut-points we found for hypertension were 25.1 kg/m^2^, followed by hypercholesterolemia and arteriosclerosis with 25.2 kg/m^2^ and hypertriglyceridemia with 25.5 kg/m^2^. The BMI cut-point for the metabolic syndrome was 26.7 kg/m^2^, for insulin resistance 27.7 kg/m^2^, for hyperinsulinemia 27.8 kg/m^2^, for increased intima media thickness 27.9 kg/m^2^, and highest for type 2 diabetes mellitus with 28.0 kg/m^2^. The ROC analysis does not indicate a strong BMI dependency for cardiovascular diseases (ROC curve not shown; AUC 0.53 [0.48–0.57]).

## 4. Discussion

To our knowledge, the BI Employee study is the first German epidemiological single-company occupational health care study. It demonstrates high prevalences of overweight and obesity in this working population. BMI was strongly associated with cardiometabolic risk factors, and their levels were significantly higher in case of overweight and obesity. Thus, also prevalence of cardiometabolic diseases was increased in overweight or obese participants, predominantly in those with male gender and ≥50 years old. The odds ratio for cardiometabolic diseases increases with each kg/m^2^, and BMI cut-points had been identified. An accumulation of nearly almost cardiometabolic diseases among overweight and obese participants was observed. In detail, highest BMI dependency was seen for the metabolic syndrome, insulin resistance and hyperinsulinemia followed by type 2 diabetes mellitus, hypertension, hypertriglyceridemia, and increased intima media thickness. Only low impact for BMI was seen for the risk of arteriosclerosis, and hypercholesterolemia, while no significant relationship was found to cardiovascular diseases.

International large-scale epidemiological cohorts of special occupational groups (i.e., civil servants in the Whitehall study [4], health care professionals in the Nurses' Health study [5], and the physicians' health study [6], etc.) have demonstrated associations between overweight/obesity and mortality risk. Nevertheless, comparable data from occupational health care cohorts from a single company are lacking. Epidemiological studies with comparable study size in this context are regional cohorts like the World Health Organization (WHO) “Monitoring Trends and Determinants in Cardiovascular Disease” (MONICA) project [7], the “Kooperative Gesundheitsforschung in der Region Augsburg” (KORA) survey [8], and the “Heinz-Nixdorf-Recall” study [9]. A national study is the “Nationale Verzehrsstudie II” (NVSII) [10] including a representative cohort of the German population (*n* = 19,329) at an age range of 40–80 years. The BI Employee study is with its study size in between those studies, and the age range of participants is comparable. With its percentage of 69% of men and 43% of women to be overweight or obese, the BI Employee study is comparable to the total population of the NVSII, where 66% of men and 51% of women had a BMI ≥ 25 kg/m^2^. However, regarding BMI categories in the corresponding age range of 40–70 years the percentage of overweight and especially of obese men (overweight: 49% versus 50–52% in the NVSII; obesity: 20% versus 20–31%) and women (overweight: 28% versus 30–38% in the NVSII; obesity: 15% versus 19–31%) in our study is at the lower range. These minor differences might have occurred due to the fact that the participants of the BI Employee study represent a working population while the population of the NVSII cohort was nationally selected, and only 54% were employed. Therefore, socioeconomic status and working-depending physical activity might have an influence on the lower prevalence of overweight and obesity.

Our results demonstrated that in case of an elevated BMI almost all cardiometabolic risk factors and diseases accumulate. However, it seems that further factors have to play together for the development of cardiovascular diseases. As a potential candidate, subclinical inflammation is discussed since increased concentrations of several inflammation markers were associated with an increased risk for incident CHD [11], unstable plaque phenotype [12], risk of coronary events and all-cause mortality [13]. Further investigations are needed to clarify which markers might have predictive potential for cardiovascular disease in our cohort.

Scientific studies from Finland [14], India [15], China [16], and USA [17] have clearly demonstrated the potential of lifestyle intervention programs on weight reduction and significant risk reduction for cardiometabolic diseases and type 2 diabetes mellitus. Thus, lifestyle interventions in the context of occupational health care with the aim of weight reduction and weight gain prevention may be essential cost-effective strategies. Our study indicates that such intervention programs should not only target overweight and obesity but also those who are not yet obese and overweight to prevent the occurrence of obesity instead of waiting for individuals to develop that stage, because at the time overweight and obesity occurred, a cluster of other risk factors and diseases has already developed. ROC analyses showed several steps of risk increase. Around 25 kg/m^2^, there are the cut-points for hypertension, hypercholesterolemia, arteriosclerosis, and hypertriglyceridemia. The more severe cardiometabolic diseases like the metabolic syndrome, insulin resistance, hyperinsulinemia, increased intima media thickness, and type 2 diabetes mellitus had their cut-point at a BMI of 27-28 kg/m^2^. Thus, operational lifestyle interventions for overweight and obese employees might be reasonable.

Strengths of the BI Employee study are clearly the study cohort of working persons in a defined age range, the big study size, the broad phenotyping, and the fact that 90% of eligible participants agreed to participate in the study. Such a high compliance is above standard and might be due to the fact that all medical services had been offered by the BI medical corporate department for free and during working time. Data from KORA demonstrated that 41% of the participants with newly diagnosed diabetes were only detected by the 2 h blood glucose measurements. In our study, type 2 diabetes mellitus might be underreported due to the fact that no oral glucose tolerance test had been performed. Here, type 2 diabetes mellitus was defined by self-report and/or fasting glucose ≥ 126 mg/dL and/or HbA1c ≥ 6.5%, therefore underestimating the true incidence. Some limitations of the results should be mentioned. This is a cross-sectional analysis of an observational study. Relationships between variables are associations and cannot be interpreted as causal. The population may not be representative for a German working population, but because of the high response rate no serious selection bias regarding the BI working population is assumed. It should be reflected that the health status might confound the ability of being and staying employed after a cardiovascular event resulting in a situation where the healthiest employees remain employed. Such bias would have led to weaker rather than stronger associations. Nevertheless, this mirrors the situation of a real working population where employees with severe illnesses already might have been retired. Adjustment to socioeconomic status had not been possible since data concerning education level, income, and occupation type had not been collected. There might be some reporting bias with respect to the end points hypertension, metabolic syndrome, insulin resistance, and arteriosclerosis because 8.6%–15.5% of these data was missing. Finally, in the definition of hypertension and hypercholesterolemia, medication was not considered.

## 5. Conclusions

The BI Employee cohort is the first large-scale epidemiological study of employees of a single company in Germany. In case of overweight and obesity nearly almost cardiometabolic risk factors and diseases accumulate and seem to represent a major problem in occupational health care. Thus, occupational health care programs for weight reduction and weight gain prevention seem to be reasonable and cost-effective strategies.

## Figures and Tables

**Table 1:** Baseline characteristics.

Parameter	Participants (*n* = 2849)	Men (*n* = 1650)	Women (*n* = 1199)
Age (years)	46.4 ± 5.9	46.5 ± 6.0	46.3 ± 5.7
Weight (kg)	80.3 ± 15.9	**87.5 ± 13.5**	70.4 ± 13.5***
BMI (kg/m^2^)	26.4 ± 4.4	**27.1 ± 3.9**	25.4 ± 4.8***
Waist circumference (cm)	94.1 ± 12.4	**98.4 ± 10.9**	88.2 ± 12.0***
Systolic blood pressure (mmHg)	130.3 ± 16.9	**133.8 ± 16.3**	125.4 ± 16.5***
Diastolic blood pressure (mmHg)	83.5 ± 9.2	**85.6 ± 8.8**	80.6 ± 9.1***
Triglycerides (mg/dL)	119.7 ± 81.9 (98)^1^	**135.8 ± 92.7 (110)** ^ 1^	97.5 ± 57.4 (81.5)^1∗∗∗^
Total cholesterol (mg/dL)	210.1 ± 36.2	**211.6 ± 36.4**	208.0 ± 35.7**
LDL cholesterol (mg/dL)	130.8 ± 33.1	**136.6 ± 32.7**	122.8 ± 31.9***
HDL cholesterol (mg/dL)	60.0 ± 16.2	**53.5 ± 12.6**	69.0 ± 16.3***
HbA1c (%)	5.4 ± 0.5	**5.5 ± 0.5**	5.4 ± 0.5***
Intima media thickness (mm)	0.7 ± 0.2	**0.7 ± 0.1**	0.6 ± 0.2***
Smoker/former smoker (%)	11/21	12/22	10/19

LDL: low-density lipoprotein; HDL: high-density lipoprotein. Shown are mean ± standard deviations. ^1^(median). There were 422–431 missings in the variables waist circumference, systolic blood pressure, diastolic blood pressure, intima media thickness. In the other variables, there were 0–55 missings. Differences between men and women had been determined by *t*-test and by Chi-square test for smoking (**P < 0.01; ***P < 0.001). Significant differences were bold written.

**Table 2:** Correlation between BMI and cardiometabolic risk factors.

Parameter	*r*
Age	0.168
Systolic blood pressure	0.394
Diastolic blood pressure	0.421
Triglycerides	0.402
Total cholesterol	0.114
LDL cholesterol	0.193
HDL cholesterol	−0.399
HbA1c	0.244
Intima media thickness	0.213

Associations had been determined by Pearson correlation (*P* < 0.0001 each).

**Table 3:** Prevalence of cardiometabolic diseases.

Parameter	Prevalence		
	All participants (*n* = 2849) [95% CI]	Stratified by sex (male/female)	Stratified by age (<50/≥50 years)
Hypertension [%]^1^	41 [39–43]	49/28***	33/58***
Hypertriglyceridemia [%]^2^	23 [21–24]	30/13***	20/28***
Hypercholesterolemia [%]^3^	62 [60–64]	65/58***	58/74***
Metabolic syndrome [%]^4^	24 [22–26]	35/9***	19/37***
Hyperinsulinemia [%]^5^	12 [11–13]	15/8***	9/18***
Insulin resistance [%]^6^	23 [21–25]	28/17***	19/33***
Type 2 diabetes mellitus [%]^7^	4 [3–5]	5/4	3/7***
Increased IMT [%]^8^	2 [2-3]	4/1***	1/6***
Arteriosclerosis [%]^9^	16 [15–18]	19/12***	9/33***
Cardiovascular disease [%]^10^	6 [5–7]	6/6	5/8**

^1^Systolic blood pressure ≥ 140 mmHg and/or diastolic blood pressure ≥ 90 mmHg or self-reported; ^2^triglycerides ≥ 150 mg/dL; ^3^total cholesterol ≥ 200 mg/dL or self-reported; ^4^waist circumference ≥ 94 cm in men, ≥80 cm in women, and two of the following criteria: hypertriglyceridemia; HDL cholesterol < 50 mg/dL in men, <40 mg/dL in women; systolic blood pressure ≥ 135 mmHg and/or diastolic blood pressure ≥ 85 mmHg; fasting glucose ≥ 100 mg/dL; ^5^fasting insulin > 15 *μ*U/mL; ^6^HOMA index ≥ 2.6; ^7^fasting glucose ≥ 126 mg/dL and/or HbA1c ≥ 6.5% and/or self-report of type 2 diabetes mellitus; ^8^intima media thickness (IMT) > 1 mm; ^9^plaques in abdominal arteries (aorta abdominalis) and/or plaques in neck arteries (aorta carotis) and/or stenoses in neck arteries (aorta carotis); ^10^Angina pectoris and/or coronary stenoses and/or myocardial infarction and/or cardiac arrhythmia and/or heart failure and/or stroke or TIA. There were 244–443 missings in the variables hypertension, metabolic syndrome, insulin resistance, increased IMT, and arteriosclerosis. In the other variables there were 7–74 missings. Differences had been determined by Chi-square test (**P < 0.01; ***P < 0.001). Significant differences were bold written.

**Table 4:** BMI-dependent risk determination.

Parameter	OR [CI]^1^ for continuous BMI	OR [CI]^2^ for BMI 25.0–29.9 kg/m^2^ BMI ≥ 30.0 kg/m^2^	Sex- and age-adjusted OR [CI]^2^ for BMI 25.0–29.9 kg/m^2^ BMI ≥ 30.0 kg/m^2^
Hypertension	**1.22 [1.19–1.25]**	**3.3 [2.7–4.0]** **8.0 [6.2–10.3]**	**2.5 [2.1–3.1]** **6.3 [4.8–8.2]**
Hypertriglyceridemia	**1.18 [1.15–1.20]**	**4.0 [3.2–5.1]** **7.4 [5.7–9.7]**	**3.2 [2.5–4.1]** **6.3 [4.8–8.3]**
Hypercholesterolemia	**1.05 [1.03–1.07]**	**1.6 [1.3–1.9]** **1.5 [1.2–1.9]**	**1.4 [1.2–1.6]** **1.3 [1.04–1.6]**
Metabolic syndrome	**1.34 [1.30–1.37]**	**15.1 [10.5–21.6]** **42.3 [28.8–62.2]**	**10.9 [7.5–15.7]** **35.9 [24.1–53.4]**
Hyperinsulinemia	**1.31 [1.28–1.35]**	**3.9 [2.6–5.9]** **22.8 [15.3–34.1]**	**3.3 [2.2–5.0]** **19.4 [12.9–29.1]**
Insulin resistance	**1.35 [1.31–1.38]**	**4.8 [3.6–6.4]** **24.7 [18.0–33.9]**	**4.1 [3.0–5.5]** **21.7 [15.8–29.8]**
Type 2 diabetes mellitus	**1.18 [1.14–1.21]**	**3.3 [1.8–6.0]** **9.3 [5.2–16.7]**	**3.0 [1.7–5.6]** **8.2 [4.5–14.9]**
Increased IMT	**1.15 [1.09–1.20]**	**2.9 [1.4–6.3]** **7.3 [3.4–15.7]**	1.6 [0.7–3.5] **3.8 [1.7–8.4]**
Arteriosclerosis	**1.07 [1.05–1.10]**	**1.9 [1.5–2.4]** **2.4 [1.8–3.2]**	**1.4 [1.03–1.8]** **1.6 [1.1–2.2]**
Cardiovascular disease	1.03 [0.99–1.06]	1.2 [0.9–1.7] 1.4 [0.9–2.2]	1.2 [0.8–1.7] 1.3 [0.9–2.0]

For definitions of parameters, see Table 3 legend. ^1^Odds ratios (ORs) and confidence intervals (CIs) were estimated corresponding to 1.0 kg/m² change for BMI. ^2^ORs were estimated versus the lowest BMI class (<25 kg/m²). ORs significantly different from 1.0 were bold written.
